# Attachment styles modulate neural markers of threat and imagery when engaging in self-criticism

**DOI:** 10.1038/s41598-020-70772-x

**Published:** 2020-08-13

**Authors:** Jeffrey J. Kim, Kirsty M. Kent, Ross Cunnington, Paul Gilbert, James N. Kirby

**Affiliations:** 1 Compassionate Mind Research Group, School of Psychology, The University of Queensland, Level 3 Building 24a, St Lucia, Brisbane, QLD 4072 Australia; 2 The Centre for Advanced Imaging, The University of Queensland, Brisbane, QLD Australia; 3 School of Allied Health and Social Care, University of Derby, Derby, DE22 1GB UK

**Keywords:** Neuroscience, Psychology

## Abstract

Attachment styles hold important downstream consequences for mental health through their contribution to the emergence of self-criticism. To date, no work has extended our understanding of the influence of attachment styles on self-criticism at a neurobiological level. Herein we investigate the relationship between self-reported attachment styles and neural markers of self-criticism using fMRI. A correlation network analysis revealed lingual gyrus activation during self-criticism, a marker of visual mental imagery, correlated with amygdala activity (threat response). It also identified that secure attachment positively correlated with lingual gyrus activation, whilst avoidant attachment was negatively correlated with lingual gyrus activation. Further, at greater levels of amygdala response, more securely attached individuals showed greater lingual gyrus activation, and more avoidantly attached individuals showed less lingual gyrus activation. Our data provide the first evidence that attachment mechanisms may modulate threat responses and mental imagery when engaging in self-criticism, which have important clinical and broader social implications.

## Introduction

A key feature of mental health difficulties is the way people develop and form internal representations of the self that then become a source of self-judgment, self-evaluation, and self-criticism. Indeed, one of the most important risk-factors for mental health difficulties is heightened and pathological self-criticism^1–3^. Self-criticism has been implicated in depression, anxiety and social phobia^4,5^, and there is substantial evidence that self-criticism can impede recovery from mental health challenges^1,6^. How self-criticism impacts, exacerbates, and impedes recovery of psychopathology has been investigated with several approaches in the literature. One line of enquiry has been the investigation of the neural correlates of self-critical processing. A review of this fMRI literature has shown that the effects of self-critical processes such as rumination recruit areas of the brain which span the medial-prefrontal and anterior cingulate cortices^7^, regions associated with mentalizing and processing salient negative events^8^. Experimentally, these findings overlap with work which has aimed to directly stimulate self-criticism during functional magnetic resonance imaging (fMRI), supporting the recruitment of these brain regions, as well as the anterior insula and amygdala^9–11^.

A second line of research has explored the association between self-critical cognitions and attachment styles, which are important individual differences that hold potential downstream consequences for psychological health. It has been proposed that self-criticism can be viewed as an outcome of insecure attachment^12^, given associations between insecure attachment styles and heightened self-criticism^13–15^. Moreover, individuals who recall their parents as critical^16^, rejecting^17^ and overprotecting^15^ are much more likely to be self-critical in contrast to those who are able to recall parental warmth^15^. Previous research has also explored neurophysiological (fMRI) responses in subjects who recalled high and low criticism from their mothers^18^. Participants who recalled their mother as critical showed greater amygdala activation and less activation of areas associated with emotional regulation, as compared with subjects who recalled low perceived criticism. This is important because how others (particularly primary attachment figures) relate to you can significantly impact how one learns to relate to oneself. That is, if your parent is overly critical of you, you are likely to internalize this process of relating and become overly critical of yourself^19^. There is also parallel evidence that early childhood maltreatment can alter emotional processing during adulthood, as assessed during fMRI^20,21^. For example, those who were mistreated as children have increased amygdala response during threat, and reduced grey matter volume in the hippocampus, insula, orbitofrontal cortex, and anterior cingulate gyrus^22^.

Whilst previous work has identified relationships between attachment styles and psychopathology, and provided neural correlates of self-criticism, such investigations have tended to be conducted in isolation. Herein we unify the two research perspectives within the literature by assessing how neural markers of self-criticism may relate to attachment style. In doing so, we anticipate our findings may provide insight into the coupling of neural markers of criticism, attachment styles and psychopathology, mechanisms that may be encoded during early childhood and could influence upon day-to-day self-relating styles and neural function during adulthood.

The fMRI paradigm used here has been reported on previously^23^. As reproduced within the present paper’s method and results, we describe how participants engaged in self-criticism versus self-reassurance, two different self-relating styles, in response to written stimuli of an emotional or neutral nature which comprise a mistake, setback or failure. Previously we reported that while engaging in self-reassurance, activation within the amygdala, anterior insula and anterior cingulate cortex were suppressed, and boosted when engaging in self-criticism. In the current paper, we re-analyze this previous data to explore the relationship between neural markers of self-criticism and self-reported attachment styles. In addition, we measured self-report variables of depression and anxiety, fears of compassion, and self-reported self-criticism which we also explored in relation to brain activation. Meta-analytic research has found significant moderate associations between these variables^24^. The aim of this paper is to examine the associations that occur between brain regions and attachment style when one is being self-critical.

## Results

The fMRI results have been reported previously^23^, and summarised again here for clarity.

### Neural activity for emotional stimuli

**Figure 1:**
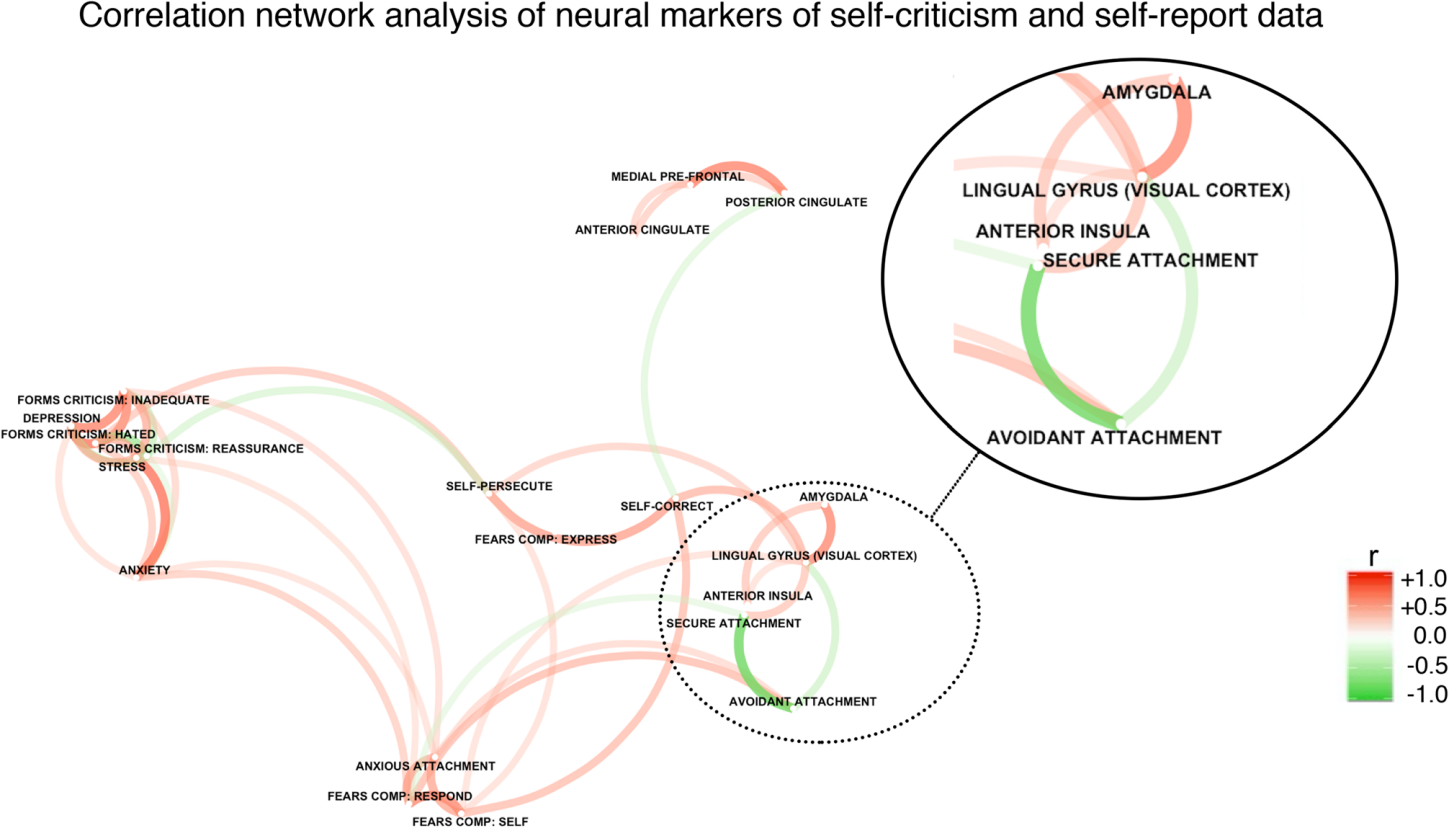
A correlation network approach which visualizes the correlation matrix between neural markers of self-criticism and self-report data. Each dot corresponds to a variable and each line corresponds to a path between correlated variables. Clusters of variables highlight constructs which are interrelated. Greater width and less transparency of a path indicates the presence of a stronger correlation. Scale bar: red colour and luminance depicts a strong positive correlation, and green colour and luminance depicts a strong negative correlation. Variable positioning is created from a multidimensional scaling of the correlation absolute values. Figure inset (solid black-lined circle) depicts the relationship between neural markers of self-criticism and self-report attachment style. *N* = 40 for all measures except attachment styles, where *N* = 38, accounting for listwise deletion. Pearson correlation formula was used.

Brain activation when participants engaged in imagery toward affective versus neutral statements was compared. A within-subjects t-contrast conducted at the group level showed significantly greater activation for emotional versus neutral statements, tested across the whole-brain, found in regions of the Default-Mode and Salience Networks, as well as portions of the occipital cortex (see Fig. 1A as reported previously^23^, all *pFWE’s* < 0.05). This activation specifically included: left Posterior Cingulate Cortex (PCC), left Calcarine Gyrus, Bilateral Lingual gyrus, left Medial Pre-frontal Cortex (MPFC), and left Anterior Cingulate Cortex (ACC) (See supplementary Table 1 as reported previously^23^ for peak co-ordinates and statistical values).

### Neural activity during self-criticism and self-reassurance

An ROI approach was conducted to assess differences in neural responses when participants engaged in the two different mental strategies, either self-criticism or self-reassurance. Peak neural responses from the ACC, MPFC, PCC and the lingual gyrus were extracted from the whole-brain contrast as reported above and in the previous paper. ROIs from the left anterior insula (AI) cortex and left amygdala were also extracted, as research from multiple studies have found associations with brain activation in these regions and compassion-related processes (as examined previously), but also self-criticism and attachment style processes^7,9–11,18,20–22^ as highlighted within the introduction of this paper. As reported previously^23^ and reproduced under a CC BY open access license, voxel-wise coordinates for each ROI were specified as each participant’s peak cluster identified with the AAL labelling tool under the WFU-pick atlas in SPM12, from the emotional-neutral contrast, for both self-criticism and self-reassurance. Each ROI is reported in XYZ coordinates in a standard space (Montreal Neurological Institute) with coordinates as follows: MPFC (2 46 36), ACC (0 14 36), PCC (4 52 36), left amygdala (− 28 − 4 − 12), left AI (− 26 10 − 14), and middle lingual gyrus (0 − 68 6). Here we focus on the correlation between these regions of interest extracted under the self-criticism condition and their relationship to self-report variables including attachment styles.

### Network correlation analysis

Following up these reults, we investigated the associations between self-reported attachment style and the brain regions implicated in self-criticism. Although we identified extensive visual cortex responses across a variety of regions, we focused on the lingual gyrus given its previous association with visual mental imagery^25,26^, as mental imagery has been identified as a potentially significant factor in self-relating^27^, in addition to ensuring consistency with our previously extracted and reported ROI. We also included additional self-report variables of depression, anxiety, and stress, fears of compassion, and self-reported self-criticism (for scale information please see methods section). To examine the potential mechanism of attachment on self-criticism, but also how these variables may interplay with our additional self-report variables, we conducted a network correlation analysis which provides a visual representation of the associations between variables (Fig. 1). Each dot and label correspond to a unique variable, and each path represents a correlation between the variables that it connects to. Positioning of each variable within the figure is created from multidimensional scaling of the absolute values of each correlation such that variables that are highly inter-correlated are depicted as clustering together. A benefit of this approach is that it allows a rich visual representation of correlations to be expressed in space. A table of means, standard deviations, scale ranges, and alpha coefficients for each scale and our regions of interest can be found in Supplementary Table 1.

### Relationship between attachment style and lingual gyrus activation

**Figure 2:**
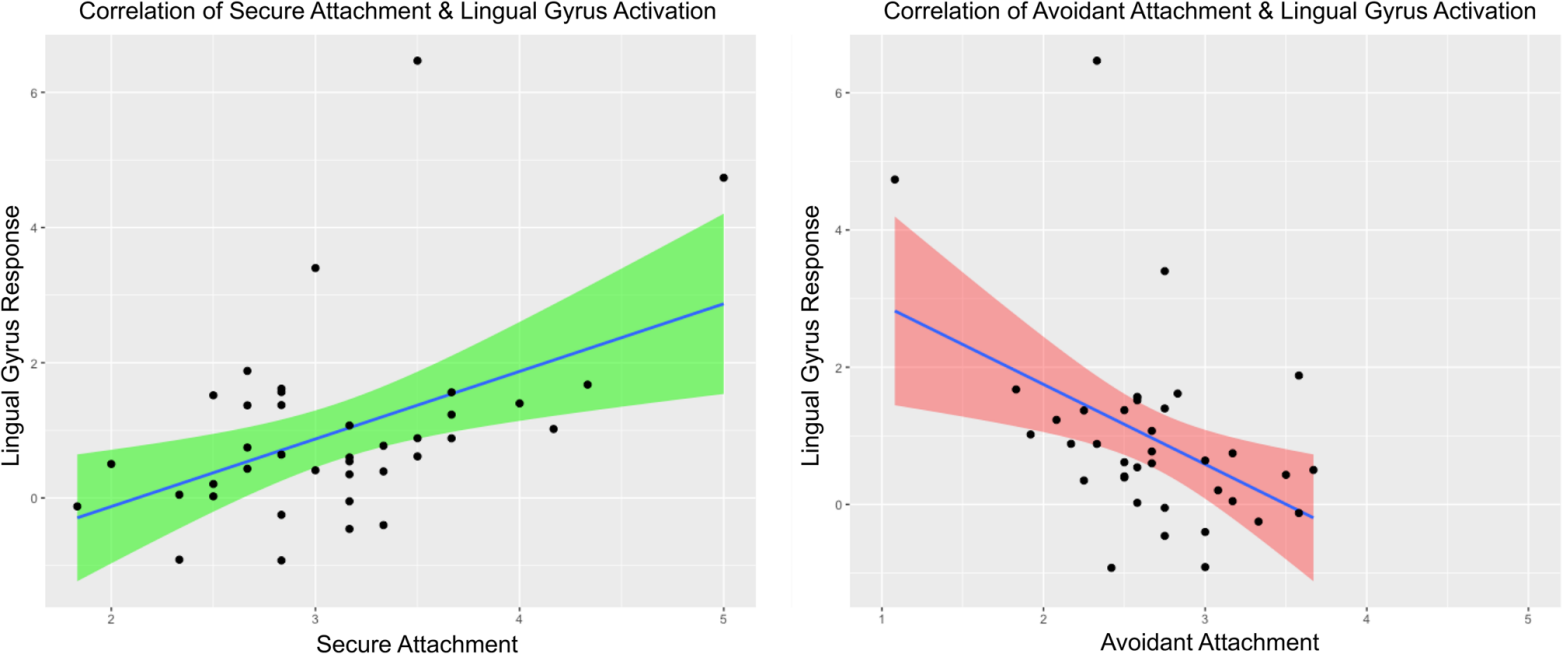
Correlations between the lingual gyrus and attachment styles, shown for **Left**. Secure and **Right**. Avoidant attachment. X-axis indicates score on the attachment subscale, and Y-axis indicates signal change of the lingual gyrus. Shaded area indicates standard error. Pearson correlation formula was used. *N* = 38, accounting for listwise deletion.

We identified a cluster within our network correlation analysis that included secure and avoidant attachment styles as well as brain regions implicated in self-criticism including the left anterior insula and left amygdala (Fig. 1 Inset). We observed that the amygdala had a particularly strong correlation to lingual gyrus activation; notable given the amygdala’s role in gating threat and fear processing, as well as its preestablished association with self-critical processes throughout the compassion and empathy literature^28,29^. Interestingly, attachment style was differentially correlated with lingual gyrus activation, with unique relationships with this brain region being shown between those with greater levels of secure attachment compared to those with greater levels of avoidant attachment. We noted that anxious attachment did not fall within this cluster, and further exploration revealed low internal consistency on this measure ($\propto \hspace{0.17em}=\hspace{0.17em}$0.49), indicating a lack of reliability. As such, we did not interpret this scale and excluded it from subsequent analyses. To explore the divergent associations between attachment styles and lingual gyrus activation, we conducted a set of correlation and moderated regression analyses examining an individual’s tendency toward secure and avoidant attachment on lingual gyrus activity. As shown in Fig. 2, our data revealed an inverse correlation pattern for more secure (*r* = 0.45, *p* = 0.004) compared to more avoidant attachment (*r* = −0.43, *p* = 0.007).

### Attachment as a moderator

**Figure 3:**
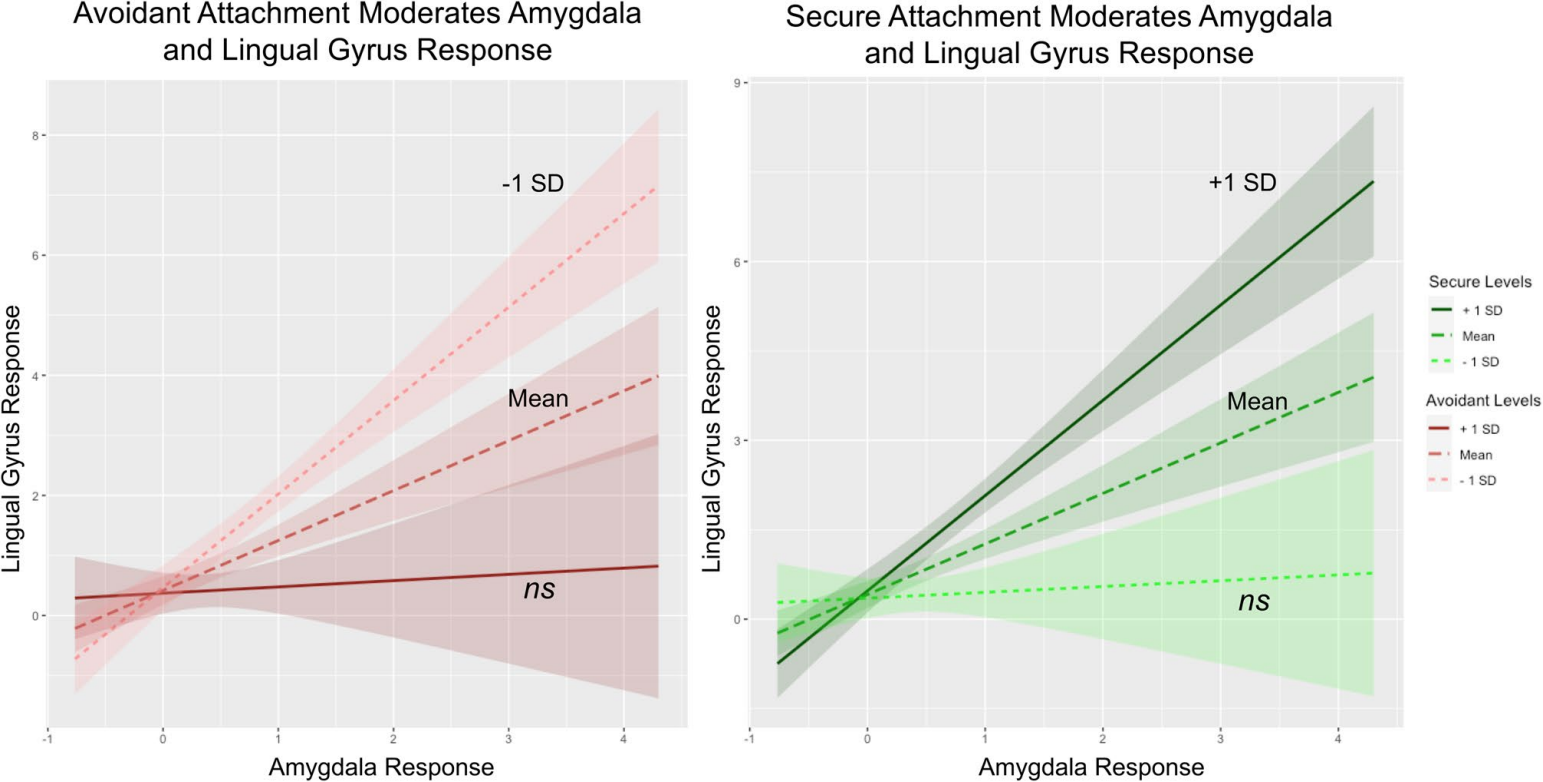
Moderation model of lingual gyrus activation for **Left.** Avoidant and **Right.** Secure attachment, at high and low levels of amygdala activation. More securely attached individuals showed higher lingual gyrus activation at greater levels of amygdala response. This effect is reversed for more avoidantly attached individuals, who showed less lingual gyrus activation at greater levels of amygdala response. Colour hues indicate mean, + 1 and − 1 *SD* of the moderator. Shaded area indicates 95% confidence interval. Figure caption denotes the shading and placement of high, mean and low levels of the moderator. Figure inset describes the mean, significant and non-significant (*ns*) simple slope for each model. *N* = 38, accounting for listwise deletion.

Given the divergent relationship between attachment styles and lingual gyrus activity shown in Fig. 2, as well as the association between lingual gyrus and amygdala activation observed in Fig. 1, we next explored how attachment styles may moderate the relationship between amygdala activation and lingual gyrus activation. A set of two hierarchical multiple regression analyses were conducted, first for secure and second for avoidant attachment styles. In the first step, two variables were included: attachment (either secure or avoidant) and amygdala activation. These variables accounted for a significant amount of variance in lingual gyrus activation in secure and avoidant attachment styles: for secure attachment, *R*^2^ = 0.55, *F*(2, 35) = 21.46, *p* < 0.001; and for avoidant attachment, *R*^2^ = 0.53, *F*(2, 35) = 19.88, *p* < 0.001. To avoid potentially problematic high multicollinearity with the interaction term, the variables were centred and an interaction term between attachment style and amygdala activation was created^30^. This interaction term was then added to the regression model, which accounted for a significant proportion of the variance in lingual gyrus activation for both secure and avoidant attachment styles: for secure attachment, Δ*R*^2^ = 0.38, Δ*F*(1, 34) = 8.02, *p* = 0.008, *b* = 0.116, *t*(34) = 0.12, *p* = 0.907; and for avoidant attachment, Δ*R*^2^ = 0.41, Δ*F*(1, 34) = 228.83, *p* < 0.001, *b* = 1.749, *t*(34) = 5.25, *p* < 0.001. Examination of the interaction plots (Fig. 3) showed that individuals with greater levels of secure attachment who were experiencing high levels of amygdala activation had greater lingual gyrus activation, whereas those with greater levels of avoidant attachment who were experiencing high levels of amygdala activation had less lingual gyrus activity. The moderation models for secure and avoidant attachment are reported in Tables 1 and 2. Follow-up simple slopes anlyses showed that these interaction effects were driven by higher levels of secure attachment (Table 3) and lower levels of avoidant attachment (Table 4).

**Table 1:** Moderation model of avoidant attachment on the relationship between amygdala and visual cortex response.

Predictor	Unstandardised B	95% CI	*t*	*sr*^2^	*R*^2^	*R*^2^(adj.)	Δ*R*^2^
Block 1	2.52	[0.69 44]	− 2.80**		0.51	0.51	0.53***
Amygdala	1.10	[0.66 1.53]	5.08***	0.35			
Avoidant attachment	− 0.79	[− 1.44 − 0.14]	− 2.47*	0.08			
Block 2	1.75	[1.07 2.43]	5.25***		0.94	0.93	0.41***
Amygdala	0.1	[− 0.09 0.32]	1.15	0.00			
Avoidant attachment	− 0.63	[− 0.87 − 0.40]	− 5.39***	0.05			
Amygdala * avoidant attachment	0.37	[0.32 0.42]	15.13***	0.41			

*N* = 38*.* CI, unstandardised B confidence intervals; DV, lingual gyrus signal change

*p < .05, **p < .01, ***p < .001.

**Table 2:** Moderation model of secure attachment on the relationship between amygdala and visual cortex response.

Predictor	Unstandardised B	95% CI	*t*	*sr*^2^	*R*^2^	*R*^2^(adj.)	Δ*R*^2^
Block 1	− 1.81	[− 3.42 − 0.20]	− 2.28*		0.55	0.53	0.55***
Amygdala	1.09	[0.67 1.52]	5.19***	0.35			
Secure attachment	0.72	[0.20 1.24]	2.80**	0.10			
Block 2	0.12	[− 1.90 2.13]	0.12		0.64	0.60	0.09**
Amygdala	− 2.8	[− 5.63 0.02]	− 2.02	0.04			
Secure attachment	0.10	[− 0.55 0.75]	0.30	0.00			
Amygdala * secure attachment	1.18	[0.33 2.03]	2.83**	0.09			

*N* = 38*.* CI, Unstandardised B confidence intervals, DV,  lingual gyrus signal change.

*p < .05, **p < .01, ***p < .001.

**Table 3:** Simple slopes analysis for secure attachment. Greater levels of secure attachment drive the moderation effect for amygdala and lingual gyrus response.

Level	Unstandardised B	SE	*t*	Moderator value	Conf. low	Conf. high
− 1 SD	0.10	0.40	0.24	2.46	− 0.72	0.91
Mean	0.85	0.21	4.02***	3.10	0.42	1.28
+ 1 SD	1.60	0.26	6.09***	3.73	1.06	2.13

DV, lingual gyrus signal change.

***p < .001.

**Table 4:** Simple slopes analysis for avoidant attachment. Lower levels of avoidant attachment drive the moderation effect for amygdala and lingual gyrus response.

Level	Unstandardised B	SE	*t*	Moderator value	Conf. low	Conf. high
− 1 SD	1.56	0.26	5.88***	2.15	1.02	2.09
Mean	0.83	0.22	3.72***	2.67	0.38	1.28
+ 1 SD	0.1	0.42	0.25	3.19	− 0.76	0.97

DV, lingual gyrus signal change.

***p < .001.

## Discussion

Here we investigated the relationship between neural markers of self-criticism and attachment styles. Our correlation network analysis identified a cluster of variables that incorporated self-report attachment styles and extracted ROIs during self-criticism which included the lingual gyrus and amygdala. We observed divergent relationships (correlations) for secure and avoidant attachment styles on lingual gyrus activation, possibly suggesting a role of attachment in visual mental imagery for self-criticism. More securely attached individuals tended to have greater lingual gyrus activation, potentially suggesting greater recruitment of mental imagery, in contrast to more avoidantly attached individuals who tended to show lower lingual gyrus activation, potentially indicating less recruitment of mental imagery. Given the divergent role of attachment styles on lingual gyrus activation in conjunction with an observed correlation between lingual gyrus activity and amygdala response, we explored whether attachment style may moderate this relationship. We found evidence for attachment styles as a moderator, whereby at greater levels of amygdala response, more securely attached individuals showed greater lingual gyrus activation, whereas individuals with lower levels of avoidant attachment exhibited greater levels of lingual gyrus activation via the amygdala.

One interpretation of the presence of attachment styles as a moderator of the relationship between neural markers of visual mental imagery and threat, is that attachment styles have implications for “inner working models” recruited under conditions of self-criticism. Specifically, securely attached individuals have access to safe attachment inner working models that provide them with the resources to cope with the threat of self-criticism^31,32^. Without the risk of threat overwhelming them, securely attached individuals are potentially more willing to engage with a threatening event and thus show a higher propensity for mental imagery during self-criticism^31,33,34^. In contrast, avoidantly attached individuals do not have access to safe attachment inner working models and therefore lack these coping resources. To protect themselves from becoming overwhelmed by the threat of self-criticism, such individuals may deploy coping strategies of denial or dissociation from the threatening event, thereby possible engaging in less mental imagery^35^.

Our results may provide further evidence for subcortical “short-cuts” for sensory and cognitive processing^36^, such as fear or threat. Previously^23^ we proposed that rapid subcortical routes for threat processing may be recruited during the act of self-criticism or self-hatred, given the brain may utilize similar routes for processing both physical and social pain^37^, but importantly from empirical work which has found an association between amygdala processing and lingual gyrus activation during threat processing^38^. Previous work has also outlined how attachment orientations can have important implications for emotional regulation and mental health^39,40^, and individual differences across attachment styles have been shown to predict divergence in neural activation and deactivation when regulating emotion^39^. We extend and unify these important ideas within the present paper, to provide empirical evidence for how visual mental imagery and the capacity for threat towards self-criticism within these regions can be modulated by attachment styles.

A broader implication of our research is the need to conceptualise self-criticism beyond a purely ‘cognitive’ process. Indeed we argue that psychological science must go beyond conceptualizing self-relating styles as a purely cognitive operation, but one that is embodied^41^. For example perhaps securely attached individuals are better able to explore and engage with their self-criticism (e.g., how self-criticism is felt within the body, experienced, and how it looks if it were to take visual form). In contrast for avoidantly attached individuals, it is also important to recognise that threat responses during self-criticism may inhibit the ability to engage in mental imagery. This points to the need to equip those with insecure attachment histories with the capacity to create their own inner working models in which they feel safe and secure^33^. In the therapeutic context this is the aim of Compassion Focused Therapy^42^. This approach includes inviting the individual to engage in compassionate mind training skills and practices, in order to build their inner sense of safeness and increase both resting and task-related physiological markers of soothing, safety and self-regulation (i.e., measured via an increase in Vagal Tone from Heart-rate Variability, HRV) which can facilitate improved threat processing^23,43–45^. Once developed, the therapy can then move to focusing on understanding the forms and functions of self-criticism, shame and trauma^46^. Based on our data, we propose that restricting therapy to only at the cognitive level of understanding self-criticism is potentially problematic.

The implications of self-relating styles for individual well-being and mental health are obvious, but the way we process and experience criticism may also have implications for our interpersonal relationships. Understanding how attachment styles may shape experiences of criticism within different role-relationships such as parenting, romantic partnership, or even organisational and leadership positions also has implications for our wellbeing and our society. The disassociation from criticism observed in those with a predominantly avoidant attachment style is likely to manifest as problematic behaviours that will affect others. For example, an avoidant parent who has internalised self-criticism but does not embody the emotional threat experience of criticism may be more likely to criticise their children, in turn leading those children to internalise patterns of self-criticism which they may pass to their own children in a vicious cycle. Similarly, romantic relationships with heightened levels of criticism are at a higher risk of collapse^47^. Avoidant attachment has also been linked to the dark triad traits of narcissism, psychopathy and Machiavellianism^48^, and our findings may suggest that an inability to embody criticism could be a common deficit amongst this cluster of traits.

A key consideration of our data, however, is that our neural and self-report attachment style links are correlational. Whilst we cannot argue causality, our work more broadly provides a greater understanding of neural markers of criticism, as well as relationships between these factors and attachment styles. In addition, a benefit of our analytic approach was to consider how attachment style can influence and attenuate present-day markers of self-criticism during fMRI, specifically by acting upon the relationship between amygdala and lingual gyrus responses. An exciting extension of our current data would be to directly assess trial-by-trial ratings of the extent of visual imagery as well as distress tolerance when participants generate self-criticism. Here, we can begin to examine how well participants are able to self-regulate threat and distress, which we would argue can be predicted from prior knowledge of attachment style, as well as physiological markers of soothing, safety and self-regulation^23^. Parallel research has investigated how compassion practices such as Compassionate Mind Training may affect neurophysiological markers of self-relating styles with measurement of Heart-rate variability^23,43,49,50^. Our current findings suggest that this research could be extended to target specific attachment styles. The present study is the first evidence that attachment style may modulate threat responses and mental imagery when engaging in self-criticism, and provides insight into how attachment mechanisms that are encoded during early childhood can potentially exhibit influence upon day-to-day self-relating styles and neural function during adulthood.

## Materials and methods

The fMRI methods have been reported previously^23^, and yet are summarised again for clarity as per a CC BY open access license.

### Participants

40 participants (Mean age = 22 years, SD = 0.49, 27 female) took part in the present study. The University of Queensland Health and Behavioural Sciences, Low and Negligible Risk Ethics Sub-Committee approved the experimental protocol, and this project complies with the provisions contained in the *National Statement of Ethical Conduct in Human Research* and complies with the regulations governing experimentation on humans. Participation was voluntary and anonymous, and subjects provided informed, written and/or electronic consent. Experimental stimuli and procedures, analysis code, data, and statistical output are available in the previous OSF repository (https://osf.io/6h9md/), and fMRI data are available on a University of Queensland Research Data Manager (RDM) server, with access available upon request.

### fMRI stimuli

We created 60 written stimuli in total, consisting of a personal mistake, setback or failure. 30 statements were of emotional valence whereas 30 were neutral (i.e., “I fail to keep up with my commitments in life”, and “I keep up with my commitments in life”, respectively). Our neutral stimuli were created to describe a non-emotive, non-intense control to counterbalance the emotional stimuli set. For both emotional and neutral sets we assessed two metrics, valence (1–5, where 1 = Very Unpleasant) and intensity (1–5, where 1 = Not Intense). Our emotional statements (*n* = 30) were revealed to be sufficiently unpleasant (*M* = 1.89) and intense (*M* = 3.54), with all neutral statements (*n* = 30) described as less unpleasant (*M* = 3.80) and comparatively not intense (*M* = 2.34).

### fMRI design

Within the scanner pre- 2-week training, we examined participant’s neural responses to the validated (affective and neutral) written stimuli when engaged in self-criticism and self-reassurance. After each trial within a block of either self-criticism or self-reassurance, participants rated how intense their degree of self-criticism or self-reassurance was to each statement (button-press on an MR-compatible button box which ranged from 1–4, where 1 = not very intense, and 4, very intense). A typical trial consisted of stimuli presented for a 6 s duration, followed by a rating of intensity for a 3 s duration, and an inter-trial-interval of 0.5 s. The first order of instruction for a particular block, that is, self-reassurance verses self-criticism, was counterbalanced for a total of 8 blocks. As our focal contrast, we manipulated the emotionality of the statements within scan runs (“emotive” vs “neutral”), in a counterbalanced order across participants. 30 statements were quasi-randomized across participants and presented for a total of 30 trials per fMRI run (~ 6.5 min total duration) over a total of 8 repeated fMRI runs. Participants were given 10 practice trials of emotional and neutral stimuli, and rated stimuli on intensity.

### fMRI acquisition and pre-processing

We collected our fMRI data on a 3-T Siemens Trio MRI scanner utilizing a 64-channel head-coil. A gradient-echo, echo-planar “fast imaging” (EPI) sequence were used to acquire functional images, with the following sequence parameters: 60 horizontal slices (2 × 2-mm in-plane voxel resolution and 2-mm slice thickness plus 10% gap), repetition time (TR) 1,000 ms; echo time (TE) 30 ms. Eight identical fMRI runs of 292 images (6 min each) were acquired. A 3D high-resolution, unified and denoised T1-weighted MP2RAGE image across the entire brain was also acquired and used as anatomical reference for subsequent pre-processing in SPM12 (TR = 4,000 ms, TE = 2.93 ms, FA = 6°, 176 cube matrix, voxel size = 1-mm). Functional imaging data were pre-processed and analyzed using SPM12, implemented in MATLAB. Structural T1-scans were co-registered to the average of the spatially realigned functional slices. Next, an inbuilt segmentation routine was applied to register each structural T1-image to the standard MNI template in MNI space. These transform parameters elicited from segmentation were subsequently applied to all realigned images, resliced to a 2 × 2 × 2-mm resolution and smoothed with 6-mm full-width-at-half-maximum (FWHM) isotropic Gaussian kernel.

### fMRI first and second-level analyses

For first-level data analysis, block-related neural responses to stimuli were modelled as 2 separate conditions (all combinations of emotional/neutral, self-criticism/self-reassurance) and convolved with the canonical hemodynamic response function (HRF). For group level analysis, whole-brain contrasts of emotional-neutral stimuli overall were reported at a cluster-level threshold of *p* < 0.05, corrected for family-wise error, with clusters formed with a voxel-level height threshold at* p* < 0.001, uncorrected. Brain regions shown to be significant had their anatomical labels identified with the Automated Anatomical Labelling (AAL) toolbox implemented in SPM12. Next, in order to examine correlations between the level of neural activation (i.e. difference in response between emotion verses neutral) and the mindset participants engaged in (i.e. self-criticism versus self-reassurance), we performed additional region of interest (ROI) analyses. For each ROI, we identified peak clusters which showed significantly greater activation overall for emotion vs neutral stimuli, and used these coordinates to extract the average contrast parameter estimates (i.e. levels of activation, Beta weights) with 5-mm radius spheres centered on those peaks for each mindset (i.e., self-criticism and self-reassurance). We then used SPSS to examine the correlation between neural responses and the mindset participants engaged in when processing neural responses to emotional stimuli. Here we focus on the relationship between self-report variables and neural markers of self-criticism via the extracted ROIs.

### fMRI trial-by-trial intensity ratings

Analysis of participant’s mean level of intensity for reassurance (emotional statements: *M* = 2.45, SD = 0.48, neutral statements: *M* = 2.63, SD = 0.64) and criticism (emotional statements: *M* = 2.92, SD = 0.45; neutral statements: *M* = 2.07, SD = 0.52) revealed intensity ratings were significantly higher for critical (emotional–neutral) but not for reassuring (emotional–neutral) trials (t(38) = 7.300, *p* < 0.001, and t(38) = −1.372, *p* = 0.178, *ns*, respectively). Inspection of a correlation matrix revealed intensity ratings for reassurance and criticism during emotional trials were correlated (R = 0.47, *p* < 0.003).

### Revised adult attachment scale

Attachment styles were measured using the Revised Adult Attachment Scale. The revised adult attachment scale measures adult attachment, partitioned into secure (“depend”), anxious, and avoidant attachment styles. In the original reporting of this scale, the Close and Depend subscales were both shown to index secure attachment^51^. Previous research has identified good validity and reliability^52^, with our sample comprising an internal consistency 0.66 for avoidant attachment, 0.72 for secure (depend) attachment, and 0.49 for anxious attachment (Supplementary Table 1).

### DASS-21

The 21-item Depression, Anxiety, and Stress Scale^53^ assesses levels of depressive, anxious and stress symptomatology across a two-week period. Greater scores on each subscale indicate greater levels of depression, anxiety and stress. Previous research has identified good validity and reliability^54^, with our sample comprising an internal consistency of 0.92 for depression subscale, 0.73 for anxiety subscale, and 0.73 for stress subscale (Supplementary Table 1).

### Fears of compassion

The Fears of Compassion Scale^55^ assesses fear of giving compassion to the self and others, as well as receiving compassion from others. Greater scores indicate greater fears of generating compassion. Previous research has identified excellent validity and reliability with this scale^24^, with our sample comprising an internal consistency of 0.85, 0.90, and 0.85 for compassion generated to self, to other, and receiving compassion, respectively (Supplementary Table 1).

### Functions of self-criticism

The functions of self-criticism scale^56^ was created to assess two competing motives for engaging in self-criticism, either self-correction (to improve the self) or self-persecution (to attack the self). Previous research has identified excellent validity and reliability^57^, with our sample comprising an internal consistency 0.79 for self-correction subscale and 0.71 for self-persecution subscale (Supplementary Table 1).

### Forms of self-criticism

The Forms of Compassion Scale^56^ assesses three distinct features of self-criticism generated toward the self; inadequacy, hatred, and reassurance. Greater scores on each subscale indicate greater levels of self-criticism. Previous research has identified good validity and reliability^58^, with our sample comprising an internal consistency of 0.89 for inadequate, 0.70 for hated, and 0.86 for reassuring forms of self-criticism, respectively (Supplementary Table 1).

## Supplementary information

Supplementary Information.

## References

[1] Werner AM, Tibubos AN, Rohrmann S, Reiss N (2019). The clinical trait self-criticism and its relation to psychopathology: a systematic review—update. J. Affect. Disord..

[2] Castilho P, Pinto-Gouveia J, Duarte J (2017). Two forms of self-criticism mediate differently the shame: psychopathological symptoms link. Psychol. Psychother. Theory, Res. Pract..

[3] Kim JJ, Gerrish R, Gilbert P, Kirby JN (2020). Stressed, depressed, and rank obsessed: individual differences in compassion and neuroticism predispose towards rank-based depressive symptomatology. Psychol. Psychother. Theory Res. Pract..

[4] Cox BJ, Fleet C, Stein MB (2004). Self-criticism and social phobia in the US national comorbidity survey. J. Affect. Disord..

[5] Cox BJ (2000). Is self-criticism unique for depression? A comparison with social phobia. J. Affect. Disord..

[6] Rose AV, Rimes KA (2018). Self-criticism self-report measures: systematic review. Psychol. Psychother. Theory Res. Pract..

[7] Nejad AB, Fossati P, Lemogne C (2013). Self-referential processing, rumination, and cortical midline structures in major depression. Front. Hum. Neurosci..

[8] Uddin LQ, Yeo BTT, Spreng RN (2019). Towards a universal taxonomy of macro-scale functional human brain networks. Brain Topogr..

[9] Doerig N (2013). Neural representation and clinically relevant moderators of individualised self-criticism in healthy subjects. Soc. Cogn. Affect. Neurosci..

[10] Longe O (2010). Having a word with yourself: neural correlates of self-criticism and self-reassurance. Neuroimage.

[11] Lutz J (2016). Altered processing of self-related emotional stimuli in mindfulness meditators. Neuroimage.

[12] Mikulincer M, Shaver PR (2007). Attachment in Adulthood: Structure, Dynamics, and Change.

[13] Brophy K, Brähler E, Hinz A, Schmidt S, Körner A (2020). The role of self-compassion in the relationship between attachment, depression, and quality of life. J. Affect. Disord..

[14] Flett GL, Burdo R, Nepon T (2020). Mattering, insecure attachment, rumination, and self-criticism in distress among university students. Int. J. Ment. Health Addict..

[15] Irons C, Gilbert P, Baldwin MW, Baccus JR, Palmer M (2006). Parental recall, attachment relating and self-attacking/self-reassurance: their relationship with depression. Br. J. Clin. Psychol..

[16] Muralidharan A, Kotwicki RJ, Cowperthwait C, Craighead W (2015). Parental relationships and behavioral approach system dysregulation in young adults with bipolar disorder. J. Clin. Psychol..

[17] Campos RC, Besser A, Blatt S (2013). Recollections of parental rejection, self-criticism and depression in suicidality. Arch. Suicide Res..

[18] Hooley JM, Siegle G, Gruber SA (2012). Affective and neural reactivity to criticism in individuals high and low on perceived criticism. PLoS ONE.

[19] Sachs-Ericsson N, Verona E, Joiner T, Preacher KJ (2006). Parental verbal abuse and the mediating role of self-criticism in adult internalizing disorders. J. Affect. Disord..

[20] Marusak HA, Martin KR, Etkin A, Thomason ME (2015). Childhood trauma exposure disrupts the automatic regulation of emotional processing. Neuropsychopharmacology.

[21] Yu M (2019). Childhood trauma history is linked to abnormal brain connectivity in major depression. Proc. Natl. Acad. Sci. USA.

[22] Dannlowski U (2012). Limbic scars: long-term consequences of childhood maltreatment revealed by functional and structural magnetic resonance imaging. Biol. Psychiatry.

[23] Kim J (2020). Neurophysiological and behavioural markers of compassion. Sci. Rep..

[24] Kirby JN, Day J, Sagar V (2019). The ‘Flow’ of compassion: a meta-analysis of the fears of compassion scales and psychological functioning. Clin. Psychol. Rev..

[25] Zhang L (2016). Gray matter volume of the lingual gyrus mediates the relationship between inhibition function and divergent thinking. Front. Psychol..

[26] de Gelder B, Tamietto M, Pegna AJ, Van den Stock J (2015). Visual imagery influences brain responses to visual stimulation in bilateral cortical blindness. Cortex.

[27] Duarte J, McEwan K, Barnes C, Gilbert P, Maratos FA (2015). Do therapeutic imagery practices affect physiological and emotional indicators of threat in high self-critics?. Psychol. Psychother. Theory Res. Pract..

[28] Kim JJ, Cunnington R, Kirby JN (2020). The neurophysiological basis of compassion: an fMRI meta-analysis of compassion and its related neural processes. Neurosci. Biobehav. Rev..

[29] Fan Y, Duncan NW, Greck MD, Northoff G (2011). Neuroscience and biobehavioral reviews is there a core neural network in empathy? An fMRI based quantitative meta-analysis. Neurosci. Biobehav. Rev..

[30] Aiken LS, West SG (1991). Multiple Regression: Testing and Interpreting Interactions.

[31] Fonagy P, Luyten P (2009). A developmental, mentalization-based approach to the understanding and treatment of borderline personality disorder. Dev. Psychopathol..

[32] Holmes J, Slade A (2018). Attachment in Therapeutic Practice.

[33] Jain FA, Fonagy P (2020). Mentalizing imagery therapy: theory and case series of imagery and mindfulness techniques to understand self and others. Mindfulness (N. Y.).

[34] Magai C, Cassidy J, Shaver PR (1999). Affect, imagery, and attachment: working models of interpersonal affect and the socialization of emotion. Handbook of Attachment: Theory, Research, and Clinical Applications.

[35] Laczkovics C (2018). Defense mechanism is predicted by attachment and mediates the maladaptive influence of insecure attachment on adolescent mental health. Curr. Psychol..

[36] Mcfadyen J, Dolan RJ, Garrido MI (2020). The influence of subcortical shortcuts on disordered sensory and cognitive processing. Nat. Rev. Neurosci..

[37] Damasio AR (2000). Subcortical and cortical brain activity during the feeling of self-generated emotions. Nat. Neurosci..

[38] Burra N (2013). Amygdala activation for eye contact despite complete cortical blindness. J. Neurosci..

[39] Mikulincer M, Shaver PR (2019). Attachment orientations and emotion regulation. Curr. Opin. Psychol..

[40] Mikulincer M, Shaver PR, Pereg D (2003). Attachment theory and affect regulation: the dynamics, development, and cognitive consequences of attachment-related strategies. Motiv. Emot..

[41] Cisek P, Kalaska JF (2010). Neural mechanisms for interacting with a world full of action choices. Annu. Rev. Neurosci..

[42] Catarino F, Gilbert P, McEwan K, Baião R (2014). Compassion motivations: distinguishing submissive compassion from genuine compassion and its association with shame, submissive behavior, depression, anxiety and stress. J. Soc. Clin. Psychol..

[43] Bello MD, Carnevali L, Petrocchi N, Thayer JF, Gilbert P, Ottaviani C (2020). The compassionate vagus: a meta-analysis on the connection between compassion and heart rate variability. Neurosci. Biobehav. Rev..

[44] Kim JJ, Parker SL, Henderson T, Kirby JN (2020). Physiological fractals: visual and statistical evidence across timescales and experimental states. J. R. Soc. Interface.

[45] Kirby JN, Doty JR, Petrocchi N, Gilbert P (2017). The current and future role of heart rate variability for assessing and training compassion. Front. Public Health.

[46] Gilbert P (2014). The origins and nature of compassion focused therapy. Br. J. Clin. Psychol..

[47] Gottman J, Gottman JM, Silver N (1995). Why Marriages Succeed or Fail: and How You Can Make Yours Last.

[48] Brewer G (2018). Dark triad traits and romantic relationship attachment, accommodation, and control. Pers. Individ. Diff..

[49] Matos M (2017). Psychological and physiological effects of compassionate mind training: a pilot randomised controlled study. Mindfulness.

[50] Barnard LK, Curry JF (2011). Self-compassion: conceptualizations, correlates, and interventions. Rev. Gen. Psychol..

[51] Collins NL, Read SJ (1990). Adult attachment, working models, and relationship quality in dating couples. J. Pers. Soc. Psychol..

[52] Ravitz P, Maunder R, Hunter J, Sthankiya B, Lancee W (2010). Adult attachment measures: a 25-year review. J. Psychosom. Res..

[53] Lovibond PF, Lovibond SH (1995). The structure of negative emotional states: comparison of the depression anxiety stress scales (dass) with the beck depression and anxiety inventories. Behav. Res. Ther..

[54] Osman A (2012). The depression anxiety stress scales-21 (DASS-21): further examination of dimensions, scale reliability, and correlates. J. Clin. Psychol..

[55] Gilbert P, Mcewan K, Matos M, Rivis A (2011). Fears of compassion: development of three self-report measures. Psychol. Psychother. Theory Res. Pract..

[56] Gilbert P, Durrant R, McEwan K (2006). Investigating relationships between perfectionism, forms and functions of self-criticism, and sensitivity to put-down. Pers. Individ. Diff..

[57] Gilbert P, Clarke M, Hempel S, Miles JNV, Irons C (2004). Criticizing and reassuring oneself: an exploration of forms, styles and reasons in female students. Br. J. Clin. Psychol..

[58] Halamová J (2018). The factor structure of the forms of self-criticising/attacking and self-reassuring scale in thirteen distinct populations. J. Psychopathol. Behav. Assess..

