# Supplementary material for: Attachment styles modulate neural markers of threat and imagery when engaging in self-criticism
Source: Sci Rep. 2020 Aug 13;10:13776. doi: 10.1038/s41598-020-70772-x (PMC7426808; doi:10.1038/s41598-020-70772-x)
Supplement: Supplementary file 1 — Supplementary Information. [file 41598_2020_70772_MOESM1_ESM.docx]

**Attachment styles modulate neural markers of threat and imagery when engaging in self-criticism**

Jeffrey J. Kim^1,2^*, Kirsty M. Kent^1^, Ross Cunnington^1^, Paul Gilbert^1,3^, & James N. Kirby^1^

^1^School of Psychology, The University of Queensland, Brisbane, Queensland, Australia

^2^The Centre for Advanced Imaging, The University of Queensland, Brisbane, Queensland, Australia

^3^School of Allied Health and Social Care, University of Derby, Derby, DE22 1GB, United Kingdom

***Corresponding Author**: Jeffrey J. Kim.

**Correspondence Address:**

School of Psychology,

Level 3 Building 24a

The University of Queensland, St Lucia, 4072

Brisbane, Qld, Australia

+61 7 3365 6230

[Jeffrey.kim@uqconnect.edu.au](mailto:Jeffrey.kim@uqconnect.edu.au)

**Keywords:** Criticism; fMRI; Salience; Vision; Attachment Style

**Author Contributions:** J.J.K, R.C, P.G, K.M.K, and J.N.K designed research; J.J.K and J.N.K. performed research; J.J.K, K.M.K, and R.C analysed data; all authors wrote the paper.

**Competing Interests:** The authors have no competing interests to declare.

**Supplementary Table 1: Self-report measures**

|  | | | | | | | | | | | | | | | | | | | | | | | | | | | | | |
| --- | --- | --- | --- | --- | --- | --- | --- | --- | --- | --- | --- | --- | --- | --- | --- | --- | --- | --- | --- | --- | --- | --- | --- | --- | --- | --- | --- | --- | --- |
|  | | **Avoidant Attachment** | | **Secure Attachment** | | **Anxious Attachment** | | **Forms Criticism: Inadequate** | | **Forms Criticism: Hated** | | **Forms Criticism: Reassuring** | | **Stress** | | **Anxiety** | | **Depression** | | **Fears: Respond Compassion** | | **Fears: Express Compassion Self** | | **Fears: Express Compass to Others** | | **Self-Correction Function of Self-Criticism** | | **Self-Persecutory Function of Self-Criticism** | |
| **Valid** |  | 38 |  | 38 |  | 38 |  | 40 |  | 40 |  | 40 |  | 40 |  | 40 |  | 40 |  | 38 |  | 38 |  | 38 |  | 38 |  | 38 |  |
| **Missing** |  | 42 |  | 42 |  | 42 |  | 40 |  | 40 |  | 40 |  | 40 |  | 40 |  | 40 |  | 42 |  | 42 |  | 42 |  | 42 |  | 42 |  |
| **Mean** |  | 2.671 |  | 3.096 |  | 2.689 |  | 2.739 |  | 1.520 |  | 3.844 |  | 11.750 |  | 10.100 |  | 9.975 |  | 27 |  | 26.63 |  | 30.71 |  | 2.850 |  | 2.214 |  |
| **Std. Deviation** |  | 0.520 |  | 0.636 |  | 0.965 |  | 0.890 |  | 0.534 |  | 0.696 |  | 3.028 |  | 3.201 |  | 4.197 |  | 6.62 |  | 7.63 |  | 10.45 |  | 0.550 |  | 0.575 |  |
| **Range** |  | 2.590 |  | 3.167 |  | 3.500 |  | 3.444 |  | 2.000 |  | 2.750 |  | 12.000 |  | 13.000 |  | 20.000 |  | 30 |  | 32 |  | 39 |  | 2.000 |  | 2.375 |  |
| **Minimum** |  | 1.080 |  | 1.833 |  | 1.170 |  | 1.222 |  | 1.000 |  | 2.125 |  | 7.000 |  | 7.000 |  | 7.000 |  | 15 |  | 16 |  | 16 |  | 1.846 |  | 1.125 |  |
| **Maximum** |  | 3.670 |  | 5.000 |  | 4.670 |  | 4.667 |  | 3.000 |  | 4.875 |  | 19.000 |  | 20.000 |  | 27.000 |  | 45 |  | 48 |  | 55 |  | 3.846 |  | 3.500 |  |
| **Alpha** |  | 0.66 |  | 0.72 |  | 0.49 |  | 0.89 |  | 0.70 |  | 0.86 |  | 0.73 |  | 0.73 |  | 0.92 |  | 0.85 |  | 0.85 |  | 0.90 |  | 0.79 |  | 0.71 |  |
|  | | | | | | | | | | | | | | | | | | | | | | | | | | | | | |

**Supplementary Table 1*.*** Descriptive statistics and internal consistency for each self-report scale included in our correlation network analysis.
